# Inherited Genetic Mutations and Polymorphisms in Malignant Mesothelioma: A Comprehensive Review

**DOI:** 10.3390/ijms21124327

**Published:** 2020-06-17

**Authors:** Vasiliki Panou, Oluf Dimitri Røe

**Affiliations:** 1 Department of Respiratory Medicine, Odense University Hospital, 5000 Odense, Denmark; 2 Department of Respiratory Medicine, Aalborg University Hospital, 9000 Aalborg, Denmark; 3 Clinical Institute, Aalborg University Hospital, 9000 Aalborg, Denmark; olufdroe@yahoo.no; 4 Department of Clinical and Molecular Medicine, Norwegian University of Science and Technology, 7491 Trondheim, Norway

**Keywords:** malignant mesothelioma, genetic cancer susceptibility, inherited genetic mutations, single nucleotide polymorphisms

## Abstract

Malignant mesothelioma (MM) is mainly caused by air-born asbestos but genetic susceptibility is also suspected to be a risk factor. Recent studies suggest an increasing number of candidate genes that may predispose to MM besides the well-characterized BRCA1-associated protein-1 gene. The aim of this review is to summarize the most important studies on germline mutations for MM. A total of 860 publications were retrieved from Scopus, PubMed and Web of Science, of which 81 met the inclusion criteria and were consider for this review. More than 50% of the genes that are reported to predispose to MM are involved in DNA repair mechanisms, and the majority of them have a role in the homologous recombination pathway. Genetic alterations in tumor suppressor genes involved in chromatin, transcription and hypoxia regulation have also been described. Furthermore, we identified several single nucleotide polymorphisms (SNPs) that may promote MM tumorigenesis as a result of an asbestos–gene interaction, including SNPs in DNA repair, carcinogen detoxification and other genes previously associated with other malignancies. The identification of inherited mutations for MM and an understanding of the underlying pathways may allow early detection and prevention of malignancies in high-risk individuals and pave the way for targeted therapies.

## 1. Introduction

Malignant mesothelioma (MM) is an aggressive tumor of the lining of the body cavities. It most often presents in the pleura, malignant pleural mesothelioma (MPM) and less often in the peritoneum, pericardium, tunica vaginalis testis and hernial sacs [1]. The histopathological subtypes are three in number, the most common epithelioid, the more rare sarcomatoid and the biphasic, that has both components [2]. MPM has a poor survival of 12–16 months for the epithelioid, and of only 4–6 months for the sarcomatoid subtype, while the five-year survival is less than 5% [3,4]. MPM is characterized by a high rate of innate and acquired chemoresistance but long-term survivors have been described both after multimodal treatment, including surgery, and chemotherapy alone [3,5,6,7]. Immunotherapy and chemoimmunotherapy are promising modalities [8]. There are no validated biomarkers that are useful for predicting the treatment response and survival in MPM.

The main cause of MPM is exposure to air-born asbestos [9]. Asbestos is a set of six minerals classified in two major groups, the amphiboles, consisting of crocidolite, amosite, tremolite, actinolite and anthophyllite, and the serpentines, namely chrysotile [9,10]. All types of asbestos are declared as carcinogens by the World Health Organization and the International Agency for Research on Cancer [9]. The latency between exposure to asbestos and MPM diagnosis has been reported to vary between 20 and 70 years [11]. Asbestos exposure can occur occupationally for asbestos workers or non-occupationally, including domestic and environmental exposure [9,12]. Asbestos was prohibited in most Western countries between 1970 and 2005, except for the USA, where it is only partly banned, and Canada, where the asbestos ban was effectuated in 2018 [13,14]. However, asbestos use and mining is ongoing in developing countries and approximately 2.2 million metric tons are being produced annually worldwide [15].

Both heavy and low-scale exposure to asbestos can cause MPM, as there is no safe threshold for asbestos use, and no linear dose–response relationship between MM and asbestos [12,16]. Nonetheless, some individuals are more susceptible to MPM subsequent to asbestos exposure than others, while there are also MM patients that report no exposure to asbestos or asbestos-like minerals [7,14]. Genetic susceptibility has long been suspected to be a risk factor for MM, providing an explanation for this observation [7]. The prevalence and spectrum of germline mutations in MM patients is not fully determined and the genetics role in causing MM de novo or enhancing asbestos carcinogenicity is yet to be ascertained. However, there have been a few published studies and case reports about genetic predisposition in MM in recent years. The aim of the current review is to summarize and present the most important studies on germline mutations that predispose to MM.

## 2. Results and Discussion

**Figure 1:**
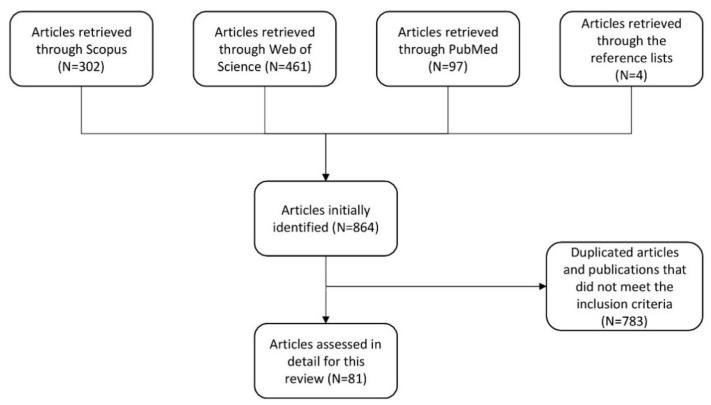
Search strategy for identifying scientific publications for this comprehensive review paper.

A total of 860 publications were retrieved through the research databases and four additional articles were identified through the reference lists. After excluding duplicated articles and publications that did not meet our inclusion criteria, there were 81 articles that were manually reviewed for this manuscript (Figure 1). The studies reporting pathogenic or likely pathogenic genetic variants and those that describe single nucleotide polymorphisms (SNPs) in genes are discussed separately due to the lower risk association of the latter with MM.

### 2.1. Pathogenic or Likely Pathogenic Genetic Variants

The most well-characterized gene that can predispose to MM is the breast cancer gene 1-associated protein 1 *(BAP1)* [17,18]. Recent studies suggest an increasing number of candidate genes associated with MM (Table 1).

#### 2.1.1. *BAP1* Gene

**Figure 2:**
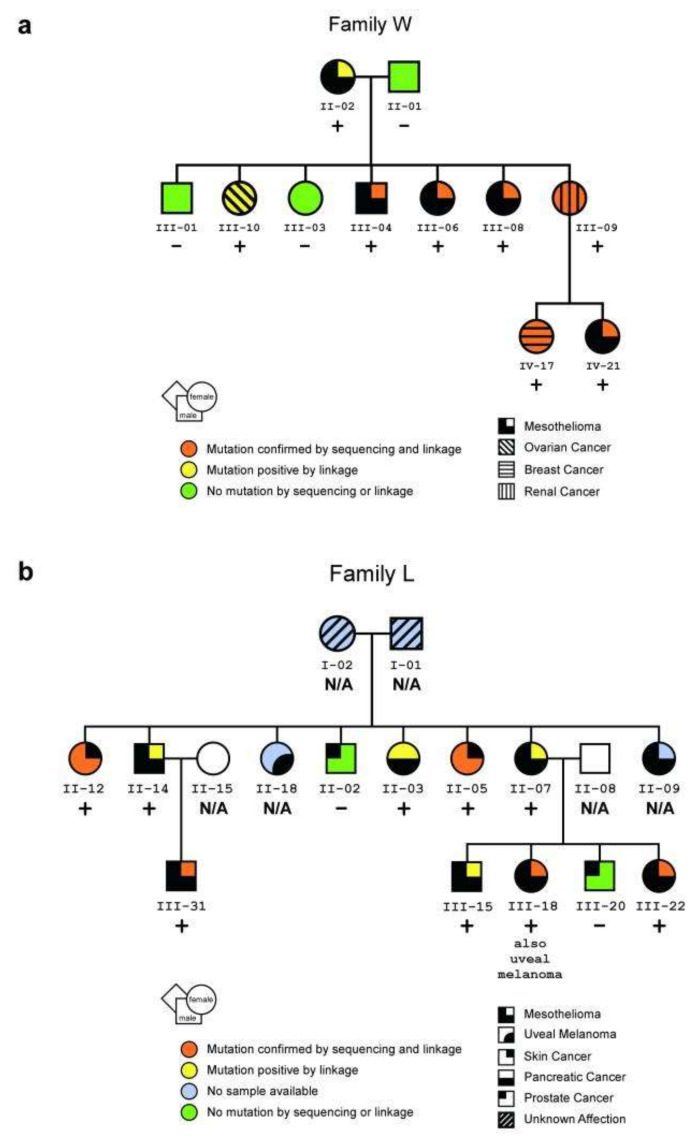
Pedigrees of two U.S. mesothelioma families. (**a**,**b**) Pedigrees showing family members with a germline mutation in *BAP1*, as confirmed by both sequencing and linkage analyses (orange) or by linkage analysis alone (yellow, i.e., no DNA was available for sequencing); individuals without the mutation (green) and individuals for whom DNA was unavailable (blue) are also shown. Presence or absence of germline *BAP1* mutation is also indicated with + or − symbols, respectively. (**a**) Pedigree of family W showing the presence or absence of a germline mutation at the *BAP1* consensus splice acceptor site. (**b**) Pedigree of family L showing the presence or absence of a germline nonsense mutation. The development of other tumor types in these families may also be related to *BAP1* germline mutations. In family W, the presence of a breast cancer before age 45 and an ovarian cancer suggests that the *BAP1* mutation is associated with a hereditary form of breast/ovarian cancer, as might be expected given *BAP1′*s relationship with the breast/ovarian cancer susceptibility gene product, BRCA115. In family L, the skin cancers shown were squamous cell carcinomas. Reprinted with permission from Springer Nature Genetics (Germline BAP1 Mutations Predispose to Malignant Mesothelioma by Testa et al.) [18], Copyright © 2012.

*BAP1* is a tumor suppressor gene, located on chromosome 3p21.1 and encodes the BAP1 nuclear protein [26]. This is a deubiquitinating hydrolase, usually part of a protein complex, participating in various cellular processes including chromatin remodeling, cell cycle regulation and growth and DNA damage response [26]. The functional roles of BAP1 are partially through its deubiquitinase activity and synergy with other proteins, such as HCFC1, YY1, OGT, ASXL1/2 and FOXK1/2, but the impact of the distinct *BAP1* mutations on the function of these complexes is not fully understood [17,27]. Germline *BAP1* mutations underlie the BAP1 tumor predisposition syndrome, associated with uveal (UM) and cutaneous melanoma (CM), MM, renal cell carcinoma (RCC), non-melanoma skin cancer, meningioma and cholangiocarcinoma as well as other cancers [18,28,29,30,31,32,33,34] (Figure 2).

*BAP1* genetic alterations appear typically with one mutant allele in all cells, while the somatic inactivation of the second allele results in tumorigenesis [17,35]. The gene–environment interaction is suspected to play an important role in cancer susceptibility for *BAP1* mutation carriers [35,36]. The pathogenic *BAP1* variants are known with a high penetration and approximately 85% of the mutation carriers are diagnosed with more than one malignancy [17,31]. Beside malignancies, individuals with germline *BAP1* mutations often present with *BAP1*-inactivated nevi, previously called melanocytic *BAP1*-mutated atypical intradermal tumors (MBAITs), that are atypical melanocytes proliferations with spitzoid morphology [26].

*BAP1* mutations are infrequent in the general population and there are no homozygotes [36,37]. However, their frequency has been reported as 1–2% for UM, 0.5% for CM and 0–7% for MM in distinct cases, rising up to 25%, 0.7% and 20%, respectively, in familial cases [4,7,36,38,39,40,41]. Patients carrying *BAP1* genetic variations were shown to have a higher incidence of peritoneal versus pleural MM [7]. In comparison with sporadic MM, the *BAP1* mutated patients tend to have sevenfold longer overall survival even when they have other cancers as well [17,42].

This is not the case in patients with other *BAP1* tumor predisposition syndrome malignancies without MM. Patients with UM and inherited *BAP1* mutations present often with a more aggressive and metastatic disease and more advanced tumor staging, and thus worse survival [43,44]. Similar findings apply to RCC and CM according to the literature [45,46,47]. The underlying molecular mechanisms that are responsible for the high variation in tumor aggressiveness in MM and the other cancers are unknown.

#### 2.1.2. Genetic Variants in DNA Repair Genes

More than half of the genes that are reported to predispose to MM are involved in DNA repair mechanisms (Table 1). The majority of the altered DNA repair genes have a role in the homologous recombination (HR) pathway, while the rest participate in the mismatch repair system (MMR), non-homologous end joining (NHEJ) or nucleotide excision repair (NER). Asbestos fibers are known to induce DNA damage, which is repaired by HR and double-strand breaks repair, MMR and NER, thus individuals with defects in the DNA repair processes are more prone to develop MM [4,7,20,23,48,49,50]. Several of the implicated genes are well-known to increase cancer susceptibility for other malignancies and many of them interact with each other.

*BRCA1* and *BRCA2* are tumor suppressors responsible to maintain genome stability, specifically in the HR pathway for double-strand DNA repair [51]. *BARD1* encodes a protein that interacts with BRCA1, forming a stable complex that is essential for tumor suppression [52]. This protein may be the target of various oncogenic mutations, for example in breast and ovarian cancer [53]. *PALB2* encodes a tumor suppressor protein that binds to BRCA2, stabilizes its localization and permits its accumulation [54]. *TP53* encodes the tumor protein p53 that determines whether DNA will be repaired or the cell will undergo apoptosis subsequent to toxic damage [55,56]. Hence, *TP53* and p53 are crucial for regulating DNA repair and cell division and genetic mutations in this gene may predispose to several malignancies [56]. *CHEK2* encodes a cell cycle checkpoint regulator and putative tumor suppressor protein, CHK2, which stabilizes p53, leading to cell cycle arrest, and interacts with BRCA1, restoring survival after DNA damage [57,58]. The protein encoded by *ATM* regulates various tumor suppressor proteins, including p53, BRCA1 and CHK2, and thus it is responsible for the cell response to DNA damage and genome stability [59]. FANCI, FANCC and FANCF are part of the Fanconi anemia complementation group (FANC) that also includes BRCA2 and PALB2 [60]. The members of the FANC group are assembled into a common protein complex that collaborate to repair DNA interstrand crosslinks after exposure to chemicals [60]. *MRE11A* encodes a protein that forms a complex with the RAD50 homolog, which is required for NHEJ and DNA double-strand break repair [61]. *XPC* encodes an important protein for NER that responds to DNA damage induced by ultraviolet radiation by recruiting ATR and ATM kinases to the DNA defect sites [62]. MLH1, MSH3, MSH6 and PSM1 are four of the seven DNA mismatch repair proteins [63]. They are necessary in order to maintain genomic stability and defects in the MMR may result in microsatellite instability and or malignant diseases, such as hereditary nonpolyposis colon cancer (HNPCC) and cancers of the NHPCC spectrum [63]. Finally, *WT1* is an oncogene that promotes HR- mediated DNA damage repair [64].

**Figure 3:**
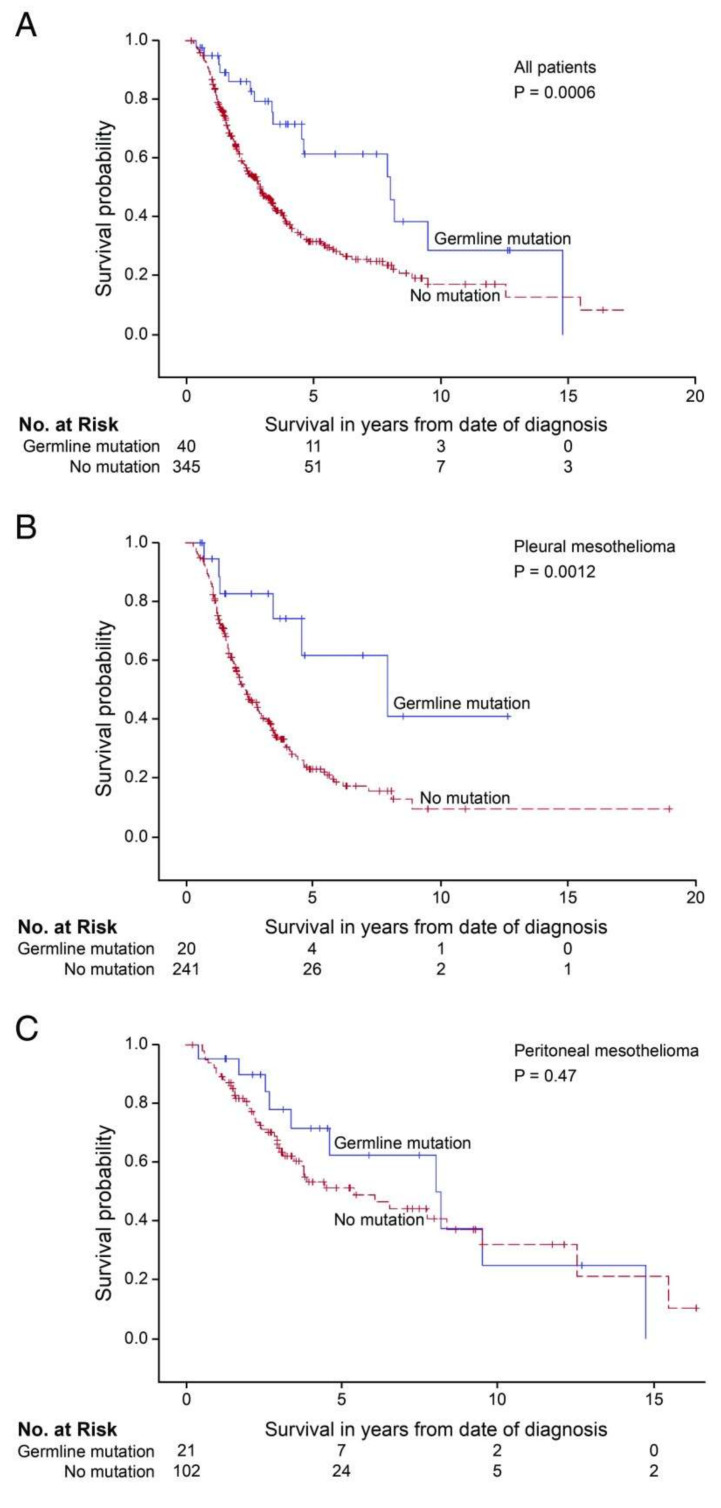
Survival of patients with mesothelioma treated with platinum-based chemotherapy, by patient’s genotype and primary site of tumor. Survival of patients with an inherited damaging mutation in any targeted gene is indicated in blue; survival of patients with no inherited mutation is indicated in red. (**A**) All mesothelioma patients with versus without inherited mutations. Median survival: 8.0 vs. 2.9 y, *p* = 0.0006. (**B**) Pleural mesothelioma patients with versus without inherited mutations. Median survival: 7.9 vs. 2.4 y, *p* = 0.0012. (**C**) Peritoneal mesothelioma patients with versus without inherited mutation. Median survival: 8.2 vs. 5.4 y, *p* = 0.47. Reprinted with permission from Proceedings of the National Academy of Sciences of the United States of America (Inherited predisposition to malignant mesothelioma and overall survival following platinum chemotherapy by Hassan et al.) [4], Copyright © 2019.

An interesting observation is that MPM patients with inherited mutations in these genes tend to have improved survival compared with those with no genetic alteration, mirroring patients with *BRCA1*- and *BRCA2*-associated malignancies [4,7,42,49] (Figure 3). Patients with ovarian, breast or prostate cancer who carry germline *BRCA1* or *BRCA2* mutations are more likely to respond to cisplatin-based chemotherapy and have better prognosis [65]. These patients have also demonstrated sensitivity to treatment with poly (ADP-ribose) polymerase inhibitors (PARPi). PARPi are proven to be effective for various solid tumors with somatic or germline mutations in HR deficit genes, including breast, prostate, ovarian and pancreatic cancer [66,67,68]. Cisplatin or carboplatin combined with pemetrexed is the cornerstone of MPM chemotherapy but a large part of the patients either do not respond or become resistant to this treatment, while there are no biomarkers in clinical use to identify potential responders [69,70]. The literature suggests that germline mutations in DNA repair and other tumor suppressor genes may be a prognostic biomarker for cisplatin chemotherapy in MPM [4,7,23]. Furthermore, there is evidence that MPM patients, especially those that are not refractory to chemotherapy, could also benefit from PARPi [4,7].

#### 2.1.3. Genetic Variants in Other Genes

A few of the genes that are reported to be mutated for MM patients are involved in chromatin regulation, including *NCOR1, ARID1A, ARID2, SMARCE1, SMARCA2* and *SMARCA4*. The protein encoded by *NCOR1* is a transcriptional coregulatory protein which assists nuclear receptors in the downregulation of gene expression [71]. ARID1A, SMARCE1, SMARCA2 and SMARCA4 are part of the ATP-dependent chromatin remodeling complex SNF/SWI, while ARID2 is a subunit of the PBAF (SWI/SNF-B) chromatin remodeling complex [72]. They are responsible for the transcriptional activation of genes that are normally repressed by chromatin and facilitate ligand-dependent transcriptional activation by nuclear receptors [72]. The SNF/SWI complexes can change the position of nucleosomes along DNA, so that binding sites for transcriptional regulators are exposed and gene expression can consequently be controlled [73,74]. Among the genes that are responsible for chromatin modifications, those encoding subunits of the SWI/SNF complexes are the most frequently mutated. Their mutations collectively occur in ∼20% of all human cancer types that have been genomically characterized so far [75]. *CREBBP* is also involved in the regulation of transcription by coupling chromatin remodeling to transcription factor recognition [76]. *SHQ1* and *RBM6* are both tumor suppressor genes involved in RNA processing, while *NF2* encodes a protein that regulates several key signaling pathways important for controlling cell shape, cell growth and cell adhesion [77,78,79]. *CDKN2A* and *TMEM127* encode both tumor suppressor proteins that are involved in cell growth, proliferation and survival [80,81]. *SMO* mediates signal transduction in the hedgehog pathway, which is critical for normal development and carcinogenesis [82]. *KDR* encodes one of the two receptors of the vascular endothelial growth factor and hereby promotes proliferation, survival, migration and differentiation of endothelial cells [83]. Finally, *VHL* and *SDHA* are involved in tumorigenesis through impaired hypoxia-inducible factor expression [84,85]. The pathophysiological mechanisms behind MM genesis as a result of these genetic alterations are not yet fully determined.

Most studies describe distinct clinical characteristics that can predict the presence of an inherited mutation, such as limited exposure to asbestos, peritoneal disease, young age and second cancer diagnosis [4,7,23,86]. This observation is of great significance, as it can lead to clinical panel-based genetic testing and the implementation of clinical genetic testing guidelines. Genetic testing would be of high benefit for MM patients and their relatives, as it would allow early detection and prevention of malignancies in high-risk individuals. This could result in the identification and treatment of the malignancies at an earlier stage, and hence improved survival. In addition, a part of these inherited mutations could be clinically significant and the patients may be able to enroll in targeted clinical trials that give them a higher chance of prolonged survival. Most importantly, the patients and their physicians should also be aware of the better survival that mutation-carriers have, as this would have a big impact on their lives and on their treatment considerations and planning.

### 2.2. Genetic Polymorphisms Associated with MM

#### 2.2.1. Genome-Wide Association Studies

Genome-wide association studies (GWAS) are the most efficient approach in detecting SNPs in MM, as they allow the simultaneous screening of thousands of genetic variants in large panels of MM patients and controls. There have been published two comprehensive GWAS regarding MM; one originating from Australia and one from Italy including 428 MM patients and 778 controls and 407 MM patients and 389 controls, respectively [87,88]. Both groups took asbestos exposure into account, as they hypothesized that MM tumorigenesis was a result of the asbestos–gene interaction. There is no compelling evidence in the two studies that the identified SNPs can cause MM in the absence of asbestos exposure. The Australian study attempted to replicate the most significant SNPs in the Italian study but failed. The heterogeneity of the populations and the different types of asbestos exposures were suggested as potential reasons for the non-replication by the researchers. The SNPs with the highest significance levels from the Australian study were located in the *CRTAM, RASGRF2* and *SDK1* genes (Table 2). All three genes are associated with cell adhesion, migration and apoptosis and they are suspected to promote carcinogenesis through mechanisms initiated by the human immune system’s response to asbestos fibers [87,89,90,91,92]. The outmost significant signals from the Italian studies were encountered in the *PVT1, ETV1, THRB, CEP350, SHC4* and *SLC7A14* genes. *PVT1, ETV1* and *THRB* are known oncogenes implicated in several malignancies through transcription regulation, such as prostate cancer, melanoma and breast cancer [88,93,94,95]. *CEP350* is required to anchor microtubules at the centrosome and *SHC4* regulates cell proliferation; their association with distinct cancers has also been reported [88,96,97]. *SLC7A14* is involved in arginine transport and although this gene has not been previously linked to MM, there are indications of neighboring genes involvement due to identified chromosomal gain in this region [88,98,99].

#### 2.2.2. Polymorphisms in Carcinogen Detoxification Genes

It is hypothesized that asbestos toxicity and carcinogenicity may be mediated by reactive oxygen species and free radicals, which occur as a result of inhaled asbestos fibers [100,101]. The four subfamilies of the glutathione S-tranferase supergene (*GSTA*, *GSTM*, *GSTT* and *GSTP*) play a central role in the detoxification and clearance of reactive oxygen species [102]. Especially the *GSTM1* and *GSTT1* subfamilies present with homozygous deletion polymorphisms (null genotype) that have been considered as modulators of susceptibility to environmentally induced malignancies [102,103]. The N-Acetyltransferase NAT2 has also an antioxidant function, as it metabolizes aromatic and heterocyclic amine carcinogens, and hence it may modify predisposition to various cancers [104]. *EPHX* encodes the epoxide hydrolase protein mEH, which is responsible for detoxification and preparation for phase II conjugation reactions [103]. Genetic polymorphisms of *EPHX* may result in an increase or decrease in enzyme activity, which may promote cancer susceptibility. Manganese superoxide dismutase (MnSOD) is an important antioxidant enzyme in mammalian tissues that is crucial for the response to reactive oxygen species [105]. The most common polymorphism of MnSOD occurs at codon 16 and results in an Alanine (Ala) to Valine amino acid transformation, which alters the protein secondary structure, and thus impairs the transport of the protein into the mitochondria [106]. *NAT2* acetylation, *EPHX*, MnSOD, *GSTM1* and *GSTT1* null polymorphisms in regard to MPM susceptibility were analyzed by Finnish and Italian researchers [100,107,108,109,110,111]. A Finnish study compared 44 MM patients to 270 controls and concluded that *GSTM1* null and the *NAT2* slow acetylator increase the MPM risk, with the heavy asbestos-exposed population being at higher risk. An Italian study comprised of 80 MPM patients and 255 healthy individuals and found similar results regarding the *GSTM1* null genotype and MPM risk. However, the *NAT2* slow acetylator appeared to be a protective factor for the Italian patients. The low-activity-associated *EPHX1* genotype was a risk factor for MM in the Italian, but not in the Finnish population. No significant risk was reported for the *GSTT1* null genotype in both studies. The nucleotidic change 282C > T within *NAT2* was also found to be significantly associated with MPM risk in another Italian study of 50 SNPs within oxidative metabolism enzymes and 75 SNPs in genome stability genes [100]. The allele 282T is connected with the haplotypes NAT2*6 and encodes for the slow acetylator phenotype [100]. Landi et al. genotyped 90 MPM patients versus 395 control subjects and found a higher MPM risk for individuals with a *GSTM1* null allele and in those with the Ala/Ala genotypes at codon 16 within MnSOD [107].

#### 2.2.3. Polymorphisms in DNA Repair Genes

Dianzani et al. focused on four DNA repair genes, *XRCC1*, *XRCC3*, *XPD* and *OGG1*, hypothesizing that deficient DNA repair mechanisms would fail to protect against the oxidative stress induced by asbestos fibers and eventually result in a higher risk of carcinogenesis [112]. *XRCC1* and *OGG1* are a part of the base excision repair (BER), while *XPD* is of the nucleotide excision repair (NER) pathway [113,114,115]. *XRRC3* participates in double-strand break repair, where *XRCC1* possibly also is involved [115]. The group investigated seven SNPs located in the four genes (i.e., *XRCC1-R399Q*, *XRCC1-R194W*, *XRCC3-T241M*, *XRCC3-IVS6-14*, *XPD-K751Q*, *XPD-D312N*, *OGG1-S326C*) which have previously been associated with various malignancies and/or impaired DNA repair [115,116,117,118,119]. The study population consisted of 81 MPM patients and 110 gender- and age-matched controls from Casale Monferrato, of which 70 patients and 85 controls had a history of asbestos exposure. Higher risk of MPM was shown for homozygotes and heterozygotes of the *XRCC1-R399Q* variant, with the risk escalating with the increasing number of *XRCC1-399Q* alleles. Further analyses were conducted after dividing the genotypes into two subgroups of ‘’risk’’ and ‘’non-risk’’ depending on the functional significance and the frequency distributions of the variants and the epidemiologic evidence. A significant association with MM was noticed for the *XRCC1-R399Q* Q homozygotes and Q/R heterozygotes versus the R homozygotes and for the *XRCC3-T241M* T homozygotes and M/T heterozygotes versus the M homozygotes. The haplotype association between two SNPs in *XRCC1, XRCC3* and *XPD* was also calculated, but significance was not reached.

## 3. Materials and Methods

Electronic searches were conducted using Scopus, PubMed and Web of Science from January 2011 to February 2020. To identify all relevant studies, we combined medical subject headings (MeSH) terms or keywords: mesothelioma AND (“gene AND mutation” OR “germline AND mutation” OR “genetic AND predisposition” OR “genetical AND predisposition” OR “genetical AND alteration” OR “genetic AND alteration” OR “germline AND alteration” OR “genetic AND susceptibility”). The reference lists of all retrieved articles were also reviewed. All publications were limited to human subjects and in the English language. Articles regarding somatic mutations were excluded, as they are out of the scope of this manuscript. Abstracts, case reports, conference presentations, editorials and expert opinions were excluded as well. All potentially relevant articles were manually reviewed.

## 4. Conclusions

There are only a few large studies investigating germline mutations and polymorphisms in MM. The first MM susceptibility gene was described in 2011, but only in the past couple of years were there published studies that shed more light into the prevalence and spectrum of germline mutations for MM. The rarity of MM together with the inadequate technologies have hampered the conduction of more comprehensive studies. The development of high-throughput technologies that allow faster and cheaper genome sequencing, such as next generation sequencing, enables researches to investigate large gene panels and identify rare genetic variants. By the use of these techniques, underlying mutations can be isolated without the requirement for lengthy genetic mapping studies. However, it is also important that the emerging data are clustered and the pathways analyzed in order to fully comprehend the underlying biological processes. Genetic testing of high-risk individuals may facilitate clinical genetic counseling and help us achieve early cancer detection. Lastly, the identification of inherited mutations and an understanding of the oncogenesis mechanisms may allow us to find potential candidates for targeted therapy, guide the choice of drug treatment and thus give MM patients a better chance of prolonged survival.

## Figures and Tables

**Table 1:** Pathogenic or likely pathogenic germline mutations associated with malignant mesothelioma*.

Gene	Location	Function	Implication in Other Tumors	Reference
*BAP1*	3	Tumor suppressor, DNA repair	Uveal and cutaneous melanoma, renal cell carcinoma, non-melanoma skin cancer, meningioma, cholangiocarcinoma	[18]
*BRCA1*	17	Tumor suppressor, DNA repair	Breast, ovarian, prostate, colon and pancreatic cancer, melanoma	[7,19]
*BRCA2*	13	Tumor suppressor, DNA repair	Breast, ovarian, prostate and pancreatic cancer, melanoma	[7,19]
*BARD1*	2	DNA repair	Breast and ovarian cancer	[20]
*TP53*	17	Tumor suppressor, DNA repair	Lung, head and neck, ovarian, breast, bladder, liver and colorectal cancer, melanoma, osteosarcoma, rhabdomyosarcoma, glioma, adrenocortical carcinoma, cholangiocarcinoma	[7,21,22]
*PALB2*	16	Tumor suppressor, DNA repair	Breast, ovarian and pancreatic cancer	[19]
*CHEK2*	22	DNA repair	Breast, ovarian and prostate cancer, osteosarcoma	[7]
*ATM*	11	DNA repair	Breast and bladder cancer, melanoma	[7,19]
*SLX4*	16	DNA repair	Head and neck and pancreatic cancer	[19]
*FANCC*	9	DNA repair	Breast, head and neck and pancreatic cancer	[19]
*FANCF*	11	DNA repair	Breast, head and neck, pancreatic and prostate cancer	[19]
*FANCI*	10	DNA repair	Breast, head and neck, pancreatic and prostate cancer	[19]
*RAD50*	5	DNA repair	Prostate and breast cancer	[23]
*MRE11A*	11	DNA repair	Breast and prostate cancer	[7]
*WT1*	11	DNA repair	Wilm´s tumor	[7]
*RECQL4*	8	DNA repair	Osteosarcoma	[20]
*XPC*	3	DNA repair	Basal and squamous cell carcinoma, melanoma	[19]
*SETD2*	3	Tumor suppressor, DNA repair, chromatin regulation	Renal cell carcinoma, leukemia	[23]
*PMS1*	2	DNA repair	Colon cancer	[19]
*MSH3*	3	DNA repair	Colon and endometrial cancer	[20]
*MSH6*	2	DNA repair	Colorectal, endometrial and ovarian cancer, leukemia, lymphoma	[7,24,25]
*MLH1*	3	Tumor suppressor, DNA repair	Colorectal, endometrial and ovarian cancer, leukemia, lymphoma	[23]
*POT1*	7	DNA repair, telomere maintenance	Melanoma, glioma	[4]
*NCOR1*	17	Chromatin regulation	-	[23]
*ARID1A*	1	Tumor suppressor, chromatin regulation	Ovarian, endometrial, kidney, stomach, bladder, lung, breast and brain cancer, cholangiocarcinoma	[23]
*SMARCE1*	17	Chromatin regulation	-	[23]
*ARID2*	12	Tumor suppressor, chromatin regulation	-	[23]
*CREBBP*	16	Tumor suppressor, transcription regulation	Bladder cancer, leukemia	[23]
*SMARCA4*	9	Tumor suppressor, chromatin regulation	Lung cancer, rhabdoid tumor predisposition syndrome type 2	[23]
*SMARCA2*	9	Tumor suppressor, chromatin regulation	Lung and head and neck cancer	[23]
*SHQ1*	3	Tumor suppressor, ribosomal and telomerase RNA processing	-	[20]
*RBM6*	3	Tumor suppressor, RNA processing	-	[23]
*NF2*	22	Tumor suppressor	Schwannoma	[21]
*CDKN2A*	9	Tumor suppressor, cell cycle regulation	Bladder, head and neck, lung, breast and pancreatic cancer, melanoma	[7,21]
*KDR*	4	Tyrosine kinase receptor	-	[23]
*TMEM127*	2	Tumor suppressor, rapamycin signaling pathway	-	[7]
*SMO*	7	G-protein couple receptor	Basal cell carcinoma	[23]
*SDHA*	5	Regulation of hypoxia inducible factor expression	Gastrointestinal stromal tumor	[7]
*VHL*	3	Regulation of hypoxia inducible factor expression	Von Hippel–Lindau syndrome	[7]

* Gene chromosome location, function and implication in other tumors were listed on the basis of gene annotations provided by the National Center for Biotechnology Information’s Online Mendelian Inheritance in Man, available online: https://www.ncbi.nlm.nih.gov/omim (accessed on 8 June 2020), The Human Gene Database, Weizmann Institute of Science, available online: https://www.genecards.org (accessed on 8 June 2020) and the Atlas of Genetics and Cytogenetics in Oncology and Haematology, available online: http://atlasgeneticsoncology.org (accessed on 8 June 2020).

**Table 2:** The most significant single nucleotide polymorphisms (SNPs) associated with malignant mesothelioma, as identified through genome-wide association studies (GWAS)*.

SNP	Locus	Gene/Neighboring Genes	Gene Function	References
rs17228032	11q24.1	*CRTAM*, *JHY*, *UBASH3B*	Adaptive immune response	[87]
rs1379270	5q13	*RASGRF2*, *CKMT2*, *MSH3*	Apoptosis, Rho and Ras protein and small GTPase mediated signal transduction regulation	[87]
rs12540101	7p22.2	*SDK1*, *CYP3A54P*, *CARD11*	Cell adhesion	[87]
rs12701229	7p22.2	*SDK1*, *CYP3A54P*, *CARD11*	Cell adhesion	[87]
rs10089418	8p21.3	*LOC286114*, *LINC02153*, *LZTS1*	-	[87]
rs11126523	2p12	*C2orf3*, *LRRTM4*	-	[87]
rs13287752	9p21.1	*MIR873*, *C9orf72*	-	[87]
rs282718	4q12	*IGFBP7*, *LINC02390*, *IGFBP7*	-	[87]
rs4707427	6q15	*SPACA1*, *AKIRIN2*	-	[87]
rs4895337	5q23.1	*FTMT*, *SRFBP1*	-	[87]
rs7958488	12p13.31	*CD27*	-	[87]
rs8142386	22q112	*LOC150185*	-	[87]
rs9548166	13q13.3	*LINC00571*, *LINC02334*	-	[87]
rs7841347	8q24.21	*PVT1*, *MYC*, *TMEM75*	Oncogene, Transcription regulation	[88]
rs3801094	7p21.2	*ETV1*, *ARL4A*, *DGKB*	Oncogene, Transcription regulation	[88]
rs9833191	3p24.2	*THRB*, *NR1D2*, *MIR4792*	Tumor suppressor, Transcription regulation	[88]
rs7632718	3q26.2	*SLC7A14*, *CLDN11*, *RPL22L1*	Amino acid transport	[88]
rs4701085	5q35.3	*ADAMTS2*, *ZNF354C*, *AX747985*	Collagen degradation	[88]
rs2501618	1q25.2	*CEP350*, *TOR1AIP1*	Microtubule anchoring	[88]
rs10519201	15q21.1	*SHC4*, *EID1*, *SEDISBP2L*	Apoptosis, Regulation of cell proliferation	[88]
rs1508805	5q23.1	*PRR16*, *FTMT*	-	[88]
rs4290865	4q22.1	*FAM190A*, *GRID2*	-	[88]
rs5756444	22q12.3	*CXF2RB2*, *C22orf33*, *TEX33*	-	[88]
rs742109	6q21	*PRDM1*, *ATGS*	-	[88]
rs9536579	13q14.3	*OLFMS*, *MIR1297*	-	[88]

* Gene location and function were listed on the basis of gene annotations provided by the National Center for Biotechnology Information’s Online Mendelian Inheritance in Man, available online: https://www.ncbi.nlm.nih.gov/omim (assessed on 8 June 2020), The Human Gene Database, Weizmann Institute of Science, available online: https://www.genecards.org (assessed on 8 June 2020) and the Ensembl genome browser, available online: https://www.ensembl.org/index.html (assessed on 8 June 2020).

## Abbreviations

MM	Malignant mesothelioma
MPM	Malignant pleural mesothelioma
SNPs	Single nucleotide polymorphisms
MeSH	Medical Subject Headings
*BAP1*	*Breast cancer gene 1-associated protein 1*
UV	Uveal melanoma
CM	Cutaneous melanoma
RCC	Renal cell carcinoma
MBAITs	Melanocytic *BAP*1-mutated atypical intradermal tumors
HR	Homologous Recombination
MMR	Mismatch repair system
NHEJ	Non-homologous recombination end joining
NER	Nucleotide excision repair
FANC	Fanconi anemia complementation group
PARPi	Poly (ADP-ribose) polymerase inhibitors
GWAS	Genome-wide association studies
